# Executive function and adult homelessness, true impairment or frontal lobology?

**DOI:** 10.3389/fnhum.2024.1359027

**Published:** 2024-01-23

**Authors:** Graham Pluck

**Affiliations:** ^1^Clinical Cognitive Sciences Laboratory, Faculty of Psychology, Chulalongkorn University, Bangkok, Thailand; ^2^Academic Clinical Psychiatry, Division of Neuroscience, University of Sheffield, Sheffield, United Kingdom

**Keywords:** homelessness, cognitive function, executive function, education, socioeconomic deprivation, frontal lobe function, addictive behaviors

## Abstract

Homelessness is associated with multiple risk factors for neurocognitive impairment. Past research with people experiencing homelessness has described “frontal lobe” dysfunction including behavioral disorders and executive cognitive impairments. In the current study, 72 adults experiencing homelessness were assessed with a standardized assessment of executive function, and interviewed regarding neurological and psychiatric history. When compared to a control sample of 25 never-homeless participants, and controlling for level of education, there was little evidence for executive dysfunction in the sample of people experiencing homelessness. Levels of substance abuse, past head injury, and post-traumatic stress disorder were notably high. However, there were no statistically significant associations between cognitive task performance and clinical or substance abuse variables. Gambling was surprisingly infrequent, but risk-taking behavior among intravenous drug users was common. Though in neither case was it linked to executive function. Overall, there was little evidence for executive impairment in this sample of people experiencing homelessness. I suggest that past research has often used inappropriate criteria for “normal” performance, particularly comparing people experiencing homelessness to control data of relatively high education level. This has led to elements of “frontal lobology,” that is, clinical neuroscience research that tends to overly link non-typical or pathological behavior to frontal lobe impairment. When appropriate comparisons are made, controlling for education level, as in this study, associations between executive function impairments and adult homelessness may be weaker than previously reported.

## 1 Introduction

Homelessness has become a substantial social and medical issue in most, if not all, developed countries, despite numerous health and social welfare services aimed at reducing its prevalence and impact (Tsai et al., 2017). There is ample clinical reason to suspect cognitive disorders would be overrepresented in populations of people experiencing homelessness. Adults experiencing homelessness report substantially raised levels of childhood abuse (Pluck et al., 2013), being victims of violence (Heerde et al., 2014), traumatic brain injury (Stubbs et al., 2020), substance abuse (Gutwinski et al., 2021), and psychotic illness (Ayano et al., 2019) which is often unmedicated (Rangu et al., 2022), amongst multiple other factors likely to impact neurocognitive functioning.

In addition to a body of literature on general neurocognitive disorder (e.g., dementia), there are multiple studies investigating focal impairments. Research on neurocognitive function of adolescents and adults experiencing homelessness has particularly focused on functions of the “frontal lobe,” despite using only behavioral measures (e.g., Pluck et al., 2015, 2018), sometimes to the extent of including the expression “frontal-lobe” or “prefrontal” in the article title, (e.g., Davidson et al., 2014; Rogoz and Burke, 2016).

Other research has reported behavioral alterations and semiology to suggest frontal lobe dysfunction in people experiencing homeless, such as neurological soft signs, disinhibition, apathy and risk-taking behavior (Douyon et al., 1998; Pluck et al., 2011; Piche et al., 2018). Top-down cognitive control, aka executive functions, abilities frequently linked to the frontal lobes (Pluck et al., 2023), have also been linked to adult homelessness (Davidson et al., 2014; Saperstein et al., 2014; Stergiopoulos et al., 2015; Hurstak et al., 2017; Fry et al., 2020; Gicas et al., 2023). Review papers have noted that, at the group level, cognitive performance of people experiencing homelessness is almost universally lower than would be expected from the general population. Furthermore, they have linked the observed impairments on executive function tests to frontal lobe disorder (e.g., Spence et al., 2004; Stone et al., 2019; Fry et al., 2020).

Several authors have suggested that frontal-lobe linked executive impairments may be contributing factors to homelessness at the individual level (Spence et al., 2004; Davidson et al., 2014; Saperstein et al., 2014; Sharma et al., 2022). Spence et al. (2004) speculated that executive control, which they linked to frontal lobe impairments, would be needed for individuals experiencing homelessness to improve their circumstances and break out of destructive behaviors. Similarly, Davidson et al. (2014) argued that the executive impairments that they observed, which they considered to be signs of prefrontal impairment, confound attempts at rehabilitation and social care of people experiencing homelessness due to potential for disadvantageous behaviors. Saperstein et al. (2014) suggested low scores on tests of executive function were predictive of inability of people experiencing homelessness to earn a wage sufficient for independent living.

This may all appear to implicate the frontal lobes in the causes and maintenance of adult homelessness. However, another way to interpret this is in what David (1992) named “frontal lobology,” that is, the tendency to link any behavior seen as non-typical or pathological to the frontal lobes of the brain. Although coined over 30 years ago, the reductionist tendency to associate complex behavioral issues with the frontal lobes remains a common phenomenon in clinical sciences dealing with the brain.

So, what else could mimic frontal-lobe impairment? An important factor is socioeconomic background, and the very closely linked issue of educational experience. Homelessness-experiencing adults are very likely to have been raised in conditions of low socioeconomic status (Koegel et al., 1995; Benjaminsen, 2016) and multiple studies have reported relatively low education levels among homelessness-experiencing populations (Fry et al., 2020; Pluck et al., 2020; Chevreau et al., 2023). This is important because neuropsychological tests of frontal-lobe behavioral traits and executive function measures are substantially affected by education, and socioeconomic background in general (Grace and Malloy, 2001; Spinella et al., 2007; Pluck et al., 2021; Pluck, 2022).

It is possible that the relatively low performance on tests of executive function, observed in multiple studies with samples of homelessness-experiencing people, is simply reflecting their socioeconomic background, rather than frontal-lobe pathology. In the current study I examined performance of adults experiencing homelessness on one of the most commonly used assessments of executive function, and a test often described as a “frontal lobe” test, the Wisconsin Card Sorting Test (WCST). However, also included are a control group matched for demographic factors. It is hypothesized that there will be no difference in task performance between homelessness-experiencing and never-homeless individuals, when education level is accounted for.

## 2 Method

### 2.1 Participants

Seventy-two homelessness-experiencing adults were recruited for the study from hostels and other services for homeless individuals in the city of Sheffield, UK. All were currently homeless based on a three criteria definition, (i) accessing services for people experiencing homelessness, (ii) lacking a permanent tenancy, and (iii) self-describing as homeless. A control group of 25 participants was recruited in the same city, with an exclusion criterion that participants had ever been homeless. An attempt was made to recruit control participants with relatively low education, as a match to the homelessness-experiencing group. Advertisements for participants in the control group were placed in community centers and welfare offices.

### 2.2 Materials

Clinical background focusing on neurological and psychiatric disorders was taken. It was not possible to consult medical notes, instead I relied on self-report. However, questions were mainly on whether the participant had ever been diagnosed with, or told by a doctor that they had, a particular disorder (regardless of whether they believed it). Interviews were performed orally, and follow-up questions were used to clarify any ambiguous responses, in an attempt to improve accuracy of the self-reports. For head injury, participants were asked if they had ever received a blow to the head that resulted in loss of consciousness for more than 30 s.

Detailed substance abuse histories were taken on past month, past year, and lifetime use for: cannabis, crack cocaine, powder cocaine, heroin, other opiates, benzodiazepines (obtained illicitly), amphetamines, ecstasy, hallucinogens, and solvents. They were also asked about intravenous drug use using the six drug-use items in the HIV Risk-Taking Behavior Scale (Darke et al., 1991). Problem alcohol use was measured with the Alcohol Use Disorders Identification Test (Saunders et al., 1993). On that scale, scored over the past 12 months, scores of 8 or greater indicate at least hazardous or harmful drinking. Pathological gambling was measured using the Gambling Inventory (Ricketts and Bliss, 2003). This also provides a classification for probable gambling based on the previous 12 months. It can be used with DSM-V criteria, in which case a probable addictive disorder would be identified with scores of 4 or more.

Clinical disorders and substance abuse were not exclusion criteria in the homelessness-experiencing group, as such disorders are so common that exclusion of individuals would produce a sample very unrepresentative of actual homeless populations. However, they were for the control sample. To measure education level of all participants, we calculated the total number of years spent in full-time formal education.

Cognitive function was assessed with the Wisconsin Card Sorting Test 64 (WCST). This standardized version of the classic test involves participants sorting each of a set of 64 cards into one of four categories, based on key cards that are provided (Kongs et al., 2000). Multiple scores can be derived from performance on the WCST, but the total number of categories achieved has the best psychometric properties in terms of reliability (Kopp et al., 2021) and validity for detecting impaired performance (Lange et al., 2018). The maximum number of possible categories achieved is 6 (higher scores indicate better performance). Normative data is available from a USA-based sample.

### 2.3 Procedure

All participants provided written informed consent, in accordance with the ethics committee approved protocol. All of the control group and some of the homelessness-experiencing participants (e.g., those who were experiencing rooflessness) were interviewed in a quiet, private room at a university hospital. The remainder of the homeless sample were interviewed in a similar office at their hostel. All assessments were performed in the morning, as participants would be less likely to be intoxicated. Any participants who confirmed that there were intoxicated were not assessed.

All interviews, including administration of the WCST, were carried out by the author, a doctoral level neuropsychologist. All participants were debriefed and given compensation for participation worth approximately US$38. Participants were also provided with pre-paid taxis to and from the interview if needed.

## 3 Results

### 3.1 Demographics

**FIGURE 1:**
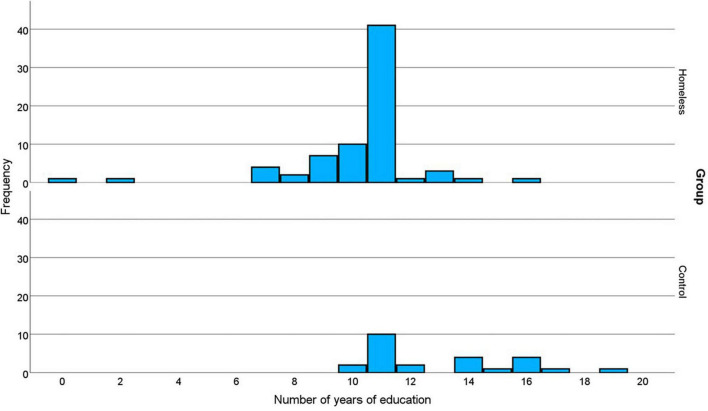
Distributions of years of education for the homelessness-experiencing and control groups.

The majority of the homelessness-experiencing group 61/72 (85%) were men, which was not significantly different to the control group (19/25 men, 76%), *X*^2^(1) = 0.98, *p* = 0.323. Similarly there was no significant difference between the groups for age, *t*(33.81) = 1.258, *p* = 0.217 (homeless mean = 35, range 18−57; control mean = 38, range 20−63). However, despite attempts to recruit control participants with relatively low educational levels, the homelessness-experiencing group had significantly fewer years of education, *t*(36.75) = 4.750, *p* < 0.001 (homeless mean = 10.3, SD = 2.2, range = 0−16; control mean = 13.0, SD = 2.6, range = 10−19). The distribution of years of education for the two groups is shown in Figure 1. For both groups the mode is 11. However, for the homelessness-experiencing group the distribution is negatively skewed, with two participants scoring very low (0 and 2 years of education). In contrast the distribution for the control group is positively skewed. Thus, although the two groups are matched on one measure of central tendency, the participants in the homelessness-experiencing group have significantly fewer years of education than those in the control group.

### 3.2 Cognitive test performance

In these analyses, to adjust for family-wise error rate, a Bonferroni correction was made for four hypotheses tested, giving an adjusted significance threshold of 0.013. The mean number of categories achieved in the WCST by the homelessness-experiencing group was 1.94 (SD = 1.50), which is lower than the control group mean of 2.72 (SD = 1.72). The data was normally distributed. A linear regression model was produced predicting the dependent variable of WCST performance with the independent variables of group and years of education. This model, summarized in Table 1, was a significant predictor of task performance. Within the model years of education was a significant predictor of task performance, but group membership was not. To test for an interaction effect, the product of those two variables was added to the model in a second stage. This increased the predictive power somewhat, but the increase was not significant.

**TABLE 1:** Regression models predicting Wisconsin Card Sorting Test performance from group membership (homeless / control) and years of education.

Predictor	*B*	*B* standard error	β	*t*	Sig.	Model fit	Sig. of change in *R*^2^
(Intercept)	−0.23	0.70		−0.33	0.74		
Group	0.28	0.40	0.08	0.70	0.49		
Education	0.18	0.07	0.30	2.70	<0.01		
						*R*^2^ = 0.12	<0.01
(Intercept)	3.87	2.33		1.66	0.10		
Group	−2.95	1.80	−0.82	−1.64	0.10		
Education	−0.17	0.20	−0.27	−0.82	0.41		
Interaction (Group*Education)	0.27	0.15	1.27	1.84	0.07		
						*R*^2^ = 0.15	0.07

Using standard measures in clinical cognitive assessment, the two groups could also be compared on cognitive performance using education-adjusted scores provided in the test manual (Kongs et al., 2000). These are used clinically to identify impairments by converting performance to percentiles. Data for the homelessness-experiencing and control groups are shown in Table 2. This method defined two-thirds of the homelessness-experiencing group as being impaired, at least mildly. However, that criterion also classified nearly half (44%) of the control sample as impaired. Nevertheless, participants in the homelessness-experiencing sample were significantly more likely to be considered impaired than participants in the control sample, *X*^2^(1,102) = 4.001, *p* = 0.045, *V* = 0.203. The qualitative interpretation of association suggests a “small” effect (Kim, 2017). Nevertheless, this seems to be over-pathologizing, given the high level of impairment suggested among the controls. If the criteria for impairment is made more stringent, at the 5th percentile, 38% of the homelessness-experiencing group meet criterion, but only 20% of the control group, but that still qualitatively small association is not significant *X*^2^(1, 102) = 2.571, *p* = 0.109, *V* = 0.163.

**TABLE 2:** Percentages of the homelessness-experiencing and control groups who scored at different percentiles for the Wisconsin Card Sorting Test (categories completed) when compared to normative data.

Percentile position	Homeless (*n* = 72)	Control (*n* = 25)	
>16^th^	33%	56%	Unimpaired
6–16^th^	29%	24%	Mild impairment
2–5^th^	21%	20%	Mild-moderate
= <1^st^	17%	0%	Moderate-severe

To summarize the results of this section, educational experience was substantially associated with performance on the WCST. When this is accounted for, there is little evidence for raised levels of impairment in the homelessness-experiencing group compared to never-homeless control group. Nevertheless, given the numerous factors that potentially could impair neurocognitive function of individuals experiencing homeless, these are explored in greater detail in the next sections.

### 3.3 Neurological, psychiatric, and forensic history

Various dichotomous measures linked to brain health are shown in Table 3. The most frequently reported medical concern was lifetime history of head injury involving unconsciousness, reported by 68% of the homelessness-experiencing group. Many of the homelessness-experiencing participants (19%) also reported past psychiatric in-patient treatment, with 6% reporting that they were legally detained for the purpose of psychiatric treatment. Almost two-thirds of the sample reported ever having been imprisoned. Considering the stigma associated with such states, the figures are likely underestimates of the true figures.

**TABLE 3:** Percentages of the homelessness-experiencing sample (*n* = 72) reporting clinical and forensic features, and the correlation with Wisconsin Card Sorting Test Performance.

Feature	Frequency	95%CI of frequency	*r*_*pb*_
Head injury	68	57−78	0.07
PTSD	32	22−43	−0.11
Seizure	18	10−28	−0.25
Epilepsy	3	0−7	n/a
Personality disorder	8	3−15	−0.09
Schizophrenia	5	1−14	−0.06
Tourette’s syndrome	1	1−4	n/a
Korsakoff’s	1	1−4	n/a
Obsessive-compulsive disorder	0	n/a	n/a
HIV	0	n/a	n/a
Psychiatric admission	19	11−29	−0.19
Legally detained for psychiatric treatment	6	1−11	−0.19
Been in prison	65	54−75	−0.07

*r*_*pb*_ values show point-biserial Pearson correlation coefficients, only calculated when frequency of feature >4. No significant associations were observed at the adjusted significance threshold of 0.007 (two-tailed).

To examine whether any of these clinical and forensic features are associated with WCST performance I examined point-biserial correlations (*r*_pb_) between each binary feature and cognitive test scores. These are shown in Table 3. In these analyses, to adjust for family-wise error rate a Bonferroni correction was made, for eight hypotheses tested, giving an adjusted significance threshold of 0.007. There were no significant associations.

To summarize this section, although clinical disorders affecting the brain were highly prevalent in the homelessness-experiencing sample, there are no statistically significant associations with WCST performance. In the final section of results, I examine how substance abuse and other addictive behaviors may be linked to executive impairment in adults experiencing homelessness.

### 3.4 Substance abuse and gambling

Levels of substance abuse in the past year were very high in the homelessness-experiencing group. Only 25/72 (35%) reported no daily use (defined as using most days over a period of at least 2 weeks). In fact, a large proportion of the sample, 28/72 (39%) had regularly used at least two different classes of substance in the past year. Looking at past month use, the most commonly abused substances were, in order, cannabis, crack cocaine, heroin, benzodiazepines, and ecstasy tablets. This is summarized in greater detail in the Supplementary Table, including correlations with WCST scores. About one-third of the homelessness-experiencing sample had been using drugs intravenously in the past month, 23/72 (32%). Of those, all showed risk-taking behaviour, e.g., reusing syringes. The mean syringe-use risk-taking score was 6.1 (SD = 4.6). There were no significant correlations between any substance abuse variables, including risky syringe use and WCST scores.

A large proportion of the homelessness-experiencing group reported no alcohol use in the past year, 28/72 (39%), however, in contrast, an even larger proportion, 31/72 (43%) were drinking at levels considered harmful or hazardous. Regarding probable gambling addiction, only 3/72 (4%) of the homelessness-experiencing participants were positive. In fact, 28/72 (39%) denied gambling at all in the past 12 months. There were no associations between any of the alcohol use or gambling addiction scores and WCST performance.

## 4 Discussion

The current results suggest that, on one widely-used measure of “frontal-lobe executive function,” there was no apparent impairment in a sample of homelessness-experiencing adults when education level is accounted for (i.e., in the linear regression). This challenges numerous studies that have suggested that executive function and other frontal-lobe related impairments are commonly observed in homelessness-experiencing people (Douyon et al., 1998; Spence et al., 2004; Pluck et al., 2011, 2015; Davidson et al., 2014; Rogoz and Burke, 2016; Piche et al., 2018; Stone et al., 2019; Fry et al., 2020). Furthermore, despite several suggestions that such deficits predispose homelessness-experiencing people to disorganized behavior and risk taking (Piche et al., 2018), we found no associations between WCST performance and two measures of risk taking. Those measures were gambling (in the full sample of homelessness-experiencing individuals) and risky HIV behavior (in the subsample who were intravenous drug users).

The reason that so many studies have linked impairments to homelessness may be partly because of comparisons of homelessness-experiencing participants to inappropriate controls. When control groups are included, rarely is their educational background matched to that of the homelessness-experiencing participants. This is important because education level is a good indicator of early-life background. Indeed, years of education is frequently used in epidemiological research as a proxy measure of an individual’s parental socioeconomic background, because it is largely influenced by caregivers and mainly fixed by adulthood (Galobardes et al., 2006). Several studies have reported either low socioeconomic status family backgrounds (Koegel et al., 1995; Benjaminsen, 2016), or low education levels in people experiencing homelessness (Fry et al., 2020; Pluck et al., 2020; Chevreau et al., 2023), which was also shown in the current study.

Because of the strong associations between cognitive test performance and socioeconomic status in general, the threshold for “impairment” is often misapplied when considering participants who come from lower education backgrounds. In contrast, two recent studies have reported cognitive function of adults experiencing homelessness, analyzed at the group level, that may be in the normal range (Chevreau et al., 2023; North et al., 2023), when compared to standardized scores. Both studies included classic tests of executive function or tests of fluid ability, which are very closely linked to the concept (Martin et al., 2015). Furthermore, both studies noted that formal education and literacy levels were substantially lower than would be expected, compared to the national population.

However, normative data is still not a good solution to the problem of detecting impaired performance in lower-education-level populations. This also often grossly overdiagnoses cognitive impairment in homelessness-experiencing and other relatively low socioeconomic status populations (Pluck, 2023). This is because most commonly, the average anchor point used to define “normal,” is that of people with average level of education for the population. One example of this is the Delis-Kaplan Executive Function System (D-KEFS; Delis et al., 2001). This is probably the most widely-used executive function battery, with a normative sample of 1,750 people. However, the normative scores are not adjusted for education level. This battery has, for example, been used to demonstrate “cognitive deficits” in people experiencing homelessness (Saperstein et al., 2014). That comparing relatively-low education level individuals to such normative scores is unfair can be shown by comparing the sample for education level. In the D-KEFS, for adults aged 30−40, only 1.3% of the normative sample had education of 8 or fewer years (that is 2 participants out of the 150 tested). In the current sample 11% had that level, a 9-fold difference.

Tellingly, Gicas et al. (2023) used a battery of executive function tests, and found impairments in their homelessness-experiencing sample only when normative tables that were not education-adjusted were used (for sustained attention and mental flexibility). When the Stroop test was analyzed, which did have education-adjusted norms, the homelessness-experiencing sample scored normally. Even if education-adjusted norms are used, they may still over pathologize homeless populations, because there is a floor effect in the tables. As an illustration, the Frontal Systems Behavior Scale (Grace and Malloy, 2001) adjusts for education level by having separate tables for participants with 12 or fewer, and more than 12 years of education. The low-education table would have been used to calculate adjusted scores for 93% of the homelessness-experiencing sample included in this study. Scores are therefore unlikely to be adequately adjusted for education level.

A maxim in neuropsychological testing, though often overlooked, is that there is “*no such thing as a neuropsychological test. Only the method of drawing inferences about the tests is neuropsychological*” (Walsh, 1992; p. 122). This important point was recently developed further by Turnbull (2023). The crux of the issue is that there are many reasons why people can perform poorly or well on a test, other than integrity of the presumed cognitive process (e.g., motivation, distractibility, past familiarity with the test materials, education level etc.). However, as Poldrack et al. (2011) have pointed out, there is a tendency within cognitive neurosciences, though quite erroneous, to equate tasks with cognitive constructs, such as referring to the “Stroop inhibition task” or “Wisconsin Card Sorting test of switching.” Hence, relatively low performance on such tasks is implicitly associated with impairment in the presumed construct.

This is likely one reason why there has been so much over-detection of cognitive impairment in homelessness-experiencing samples. The WCST has been so widely used to detect supposed frontal lobe impairments that it is often referred to explicitly as the Wisconsin Card Sorting Test of frontal lobe integrity (e.g., Clark et al., 2005; Chamberlain et al., 2006) or other such names explicitly labelling it as a measure of frontal function. Thus, researchers can sometimes erroneously assume that low scores on the test indicate frontal lobe impairments, neglecting the overall context of performance, such as education level of the test taker.

This bias led David (1992) to highlight what he called “psychiatry’s new pseudoscience,” jokingly naming it “frontal lobology.” One of the issues that David raised was the specificity of measures of “frontal lobe function” such as the WCST. Even in the neurologically healthy, relatively low performance on such tests is influenced by a range of factors. In fact, impaired performance on the WCST is just as likely after posterior brain lesions as it is after frontal lobe lesions (Jodzio and Biechowska, 2010), and to consider it a pure test of frontal function is highly inaccurate (Nyhus and Barcelo, 2009). Overall, it is not reasonable to assume that performance on that test can reveal much about integrity of the frontal lobes specifically, though, if interpreted carefully, it can be used to infer cerebral impairment. The same rule can be applied to several other cognitive tests that have been used to infer frontal lobe impairments in homelessness-experiencing samples, such as the Trail Making Test (Rogoz and Burke, 2016), which is also not specifically sensitive to frontal lobe dysfunction (Chan et al., 2015).

I have, as a researcher, shown this bias in my own studies on homelessness. The issue of frontal lobology is being raised here not to accuse any researchers of pseudoscience, but to bring awareness of the risks of over pathologizing. This can have serious consequences, especially when it involves an already very marginalized demographic, such as people who are experiencing homelessness.

Furthermore, the negative result found here, when education level is controlled for, certainly cannot rule out some level of cognitive impairment associated with homelessness. Given the multiplex physical and psychological health challenges faced by many people lacking homes, there often will be some impact on neurocognition. Nevertheless, the severity of this may have been exaggerated. In the current study I report high levels of substance abuse, neurological, and psychological illness. However, of multiple factors examined, none were significantly associated with WCST performance. Many previous studies have linked these factors to neurocognitive performance in non-homeless samples. It is perhaps, because of the multiple pathways to homelessness that these factors are not strongly associated. For example, although substance abuse may impair cognition in some people who are experiencing homelessness, it may also be that some people with relatively higher levels of cognitive ability become homeless due to their substance use. This would obscure simple linear relationships between substance abuse and cognitive ability. Similar issues could be involved with neurological and psychiatric illnesses.

One final observation, again, against the conclusion of a general frontal-lobe syndrome associated with homelessness, is the very low level of pathological gambling reported by the homelessness-experiencing sample. Despite high levels of other addictive behaviors, such as intravenous substance abuse, only 4% of the homelessness-experiencing sample reported pathological gambling. Such behavior is often associated with idiopathic and acquired neurological disorder affecting frontal-subcortical circuits (Santangelo et al., 2013; Turner et al., 2019), and many pathological gamblers show executive function deficits. The low prevalence reported here is therefore not consistent with a dysexecutive syndrome linked to homelessness.

In conclusion, little evidence is provided in the current research to support an executive function impairment associated with adult homelessness. It is suggested that “frontal lobe syndrome,” linked to homelessness in many previous studies, is overestimated due to misleading comparisons of neuropsychological test scores to inappropriate control groups or normative data.

## Data availability statement

The raw data supporting the conclusions of this article will be made available by the authors, without undue reservation.

## Ethics statement

The studies involving humans were approved by the North Sheffield Research Ethics Committee. The studies were conducted in accordance with the local legislation and institutional requirements. The participants provided their written informed consent to participate in this study.

## Author contributions

GP: Conceptualization, Data curation, Formal analysis, Investigation, Methodology, Writing – original draft, Writing – review & editing.
