# Supplementary material for: Executive function and adult homelessness, true impairment or frontal lobology?
Source: Front Hum Neurosci. 2024 Jan 23;18:1359027. doi: 10.3389/fnhum.2024.1359027 (PMC10844389; doi:10.3389/fnhum.2024.1359027)
Supplement: Supplementary file 1 [file Table_1.DOCX]

Supplementary material

Table: Frequency of the five most commonly abused substances by the homelessness-experiencing sample in the past month, shown as percentage of sample, also shown is the correlation of use frequency with Wisconsin Card Sorting Test scores

| Substance | None | Once | Occasional | Daily | Any use | *r* |
| --- | --- | --- | --- | --- | --- | --- |
| Cannabis | 40 | 1 | 29 | 29 | 60 | .07 |
| Crack cocaine | 60 | 4 | 15 | 21 | 40 | .11 |
| Heroin | 67 | 3 | 6 | 25 | 33 | .15 |
| Benzodiazepine | 78 | 7 | 8 | 7 | 22 | -.13 |
| Ecstasy | 86 | 7 | 7 | 0 | 14 | .17 |

*r* values show Pearson correlation coefficients, none were significant at the Bonferroni adjusted significance threshold of *p* < .001 (two-tailed). Some sets do not sum to 100 because of rounding effects when calculating the percentages.
